# Recent advances in transcatheter management of pulmonary regurgitation after surgical repair of tetralogy of Fallot

**DOI:** 10.12688/f1000research.14301.1

**Published:** 2018-05-30

**Authors:** Matthew I Jones, Shakeel A Qureshi

**Affiliations:** 1 Evelina London Children’s Hospital, Guy’s & St Thomas’ NHS Foundation Trust, Westminster Bridge Road, London, SE1 7EH, UK

**Keywords:** tetralogy of Fallot, pulmonary regurgitation, transcatheter

## Abstract

Surgical repair of tetralogy of Fallot (ToF) in childhood is associated with generally good outcomes, and almost all children can be expected to survive until adulthood. However, significant pulmonary regurgitation leading to progressive right ventricular dilatation is common in teenagers or young adults because of the nature of the surgical intervention. In patients whose repair included placement of a right ventricle to pulmonary artery conduit, it has been possible to place a stented valve within the conduit to treat this. Pulmonary regurgitation after repair of ToF via a transannular patch technique has historically involved repeat surgery as the dimensions of the right ventricular outflow tract have been too large for commercially available valves. This review summarises the novel transcatheter valves available for management of pulmonary regurgitation after surgical repair of ToF in patients in whom the dimensions of the right ventricular outflow tract have previously been considered too large for transcatheter valve implantation.

## Introduction

Tetralogy of Fallot (ToF) is one of the commoner forms of congenital heart disease, accounting for up to 10% of all children with congenital heart defects. Results from surgical correction of the defect are generally good, and more than 95% of children undergoing repair can be expected to survive to adulthood. However, surgical right ventricular outflow tract (RVOT) enlargement, often combined with a transannular patch to abolish obstruction completely, results in varying degrees of pulmonary valve regurgitation. Severe pulmonary regurgitation can result in progressive dilatation and dysfunction of the right ventricle, decrease in exercise tolerance, arrhythmias, right heart failure and increased risk of sudden death ^1^. Historically, most children and young adults in whom there was felt to be an indication for implantation of a competent pulmonary valve were referred for surgery. However, transcatheter valve implantation became a reality in the year 2000, when Bonhoeffer and colleagues implanted the first percutaneous pulmonary valve ^2^. Initially, these valves were implanted into an *in situ* fixed-dimension conduit, but, with time, the spectrum of anatomy for which transcatheter valves could be used grew to the point where most centres would now consider a percutaneous approach to the management of pulmonary regurgitation to be the first-line therapy. However, use of the commercially available transcatheter valves—the Melody valve (Medtronic, Minneapolis, MN, USA) and the SAPIEN series of valves (Edwards Lifesciences, Irvine, CA, USA)—is limited by their maximal dimensions and the fact that the majority of patients who have undergone ToF repair via a transannular patch technique have RVOT dimensions larger than those suitable for either of these valves. This review summarises the novel transcatheter valves that are in clinical trials at varying stages and touches briefly on hybrid surgical and transcatheter approaches to pulmonary valve implantation.

## Venus P-valve

**Figure 1.**
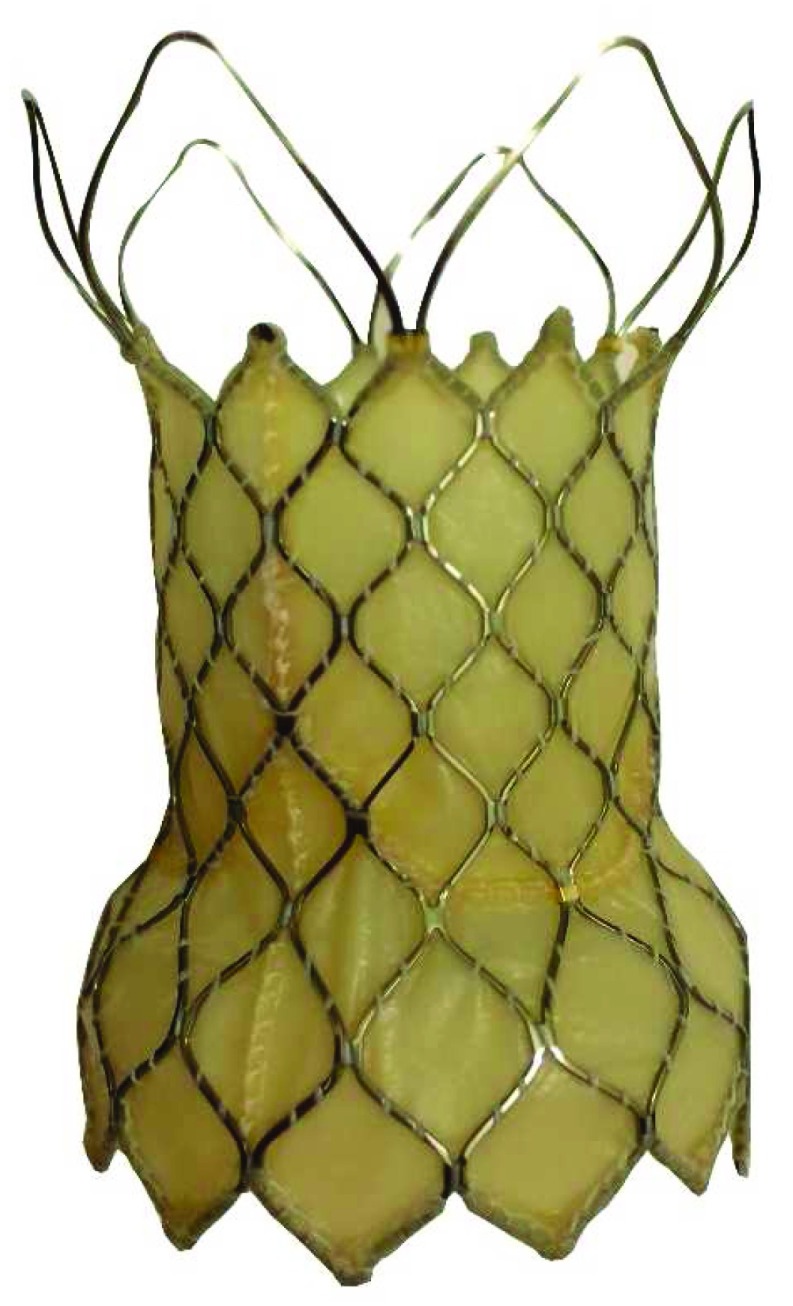
Venus P-valve. The Venus P-valve (Venus MedTech, Shanghai, China) has proximal and distal flares that anchor the valve in the right ventricular outflow tract. The distal flare is not covered, permitting unobstructed flow into the branch pulmonary arteries.

The Venus P-valve (Venus MedTech, Shanghai, China) is a self-expanding percutaneous valve comprising a nitinol stent and a tri-leaflet porcine pericardial tissue valve hand-sewn inside the nitinol frame and designed to be implanted into a patch-reconstructed RVOT ( Figure 1). At present, it has not received CE (Conformité Européene) certification and has not been approved for use by the US Food and Drug Administration, although a CE feasibility and safety study is being undertaken at our institution, amongst others.

**Figure 2.**
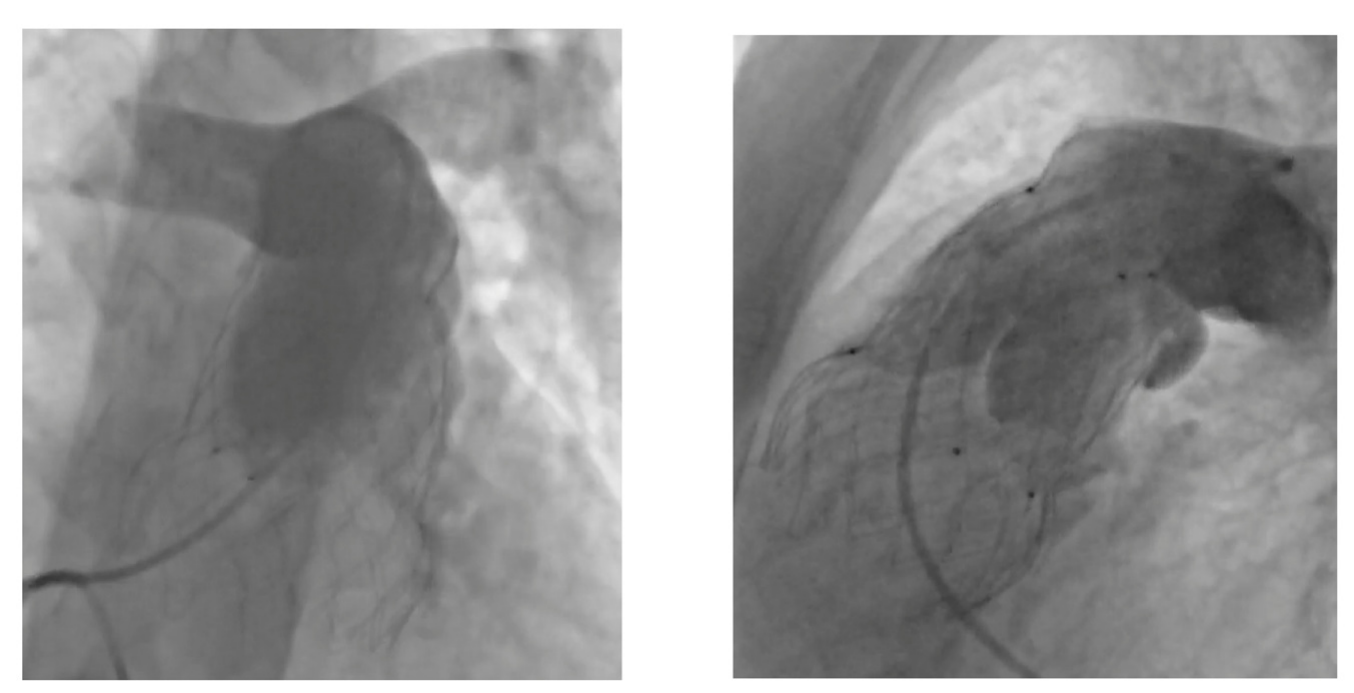
Angiographic appearances of the Venus P-valve after deployment as seen in the left anterior oblique/cranial and lateral projections. Here, the Venus P-valve (Venus MedTech, Shanghai, China) is at level of the proximal markers, immediately above the right ventricular outflow tract.

Early clinical experience in five patients with severe pulmonary regurgitation following surgical repair of ToF via a transannular patch was promising with excellent procedural success and good immediate and short-term valve function ^3^. The feasibility of implanting this valve in humans was demonstrated by recent series from Asia ^4^, Europe ^5^ and South America ^6^. Each of these studies has demonstrated predictable procedural success. The flared distal portion of the stent allows anchoring at the pulmonary artery bifurcation, whilst the uncovered struts at the distal end allow unobstructed branch pulmonary artery flow ( Figure 2). The valve can be implanted in the RVOTs after transannular patch repair (or other reconstruction), whose narrowest diameter is less than 34 mm. The 34 mm narrowest diameter is suitable for the 36 mm valve. Up to the currently available larger sizes, the valve can be implanted with reliable reproducibility and good valve function.

## Harmony transcatheter pulmonary valve

**Figure 3.**
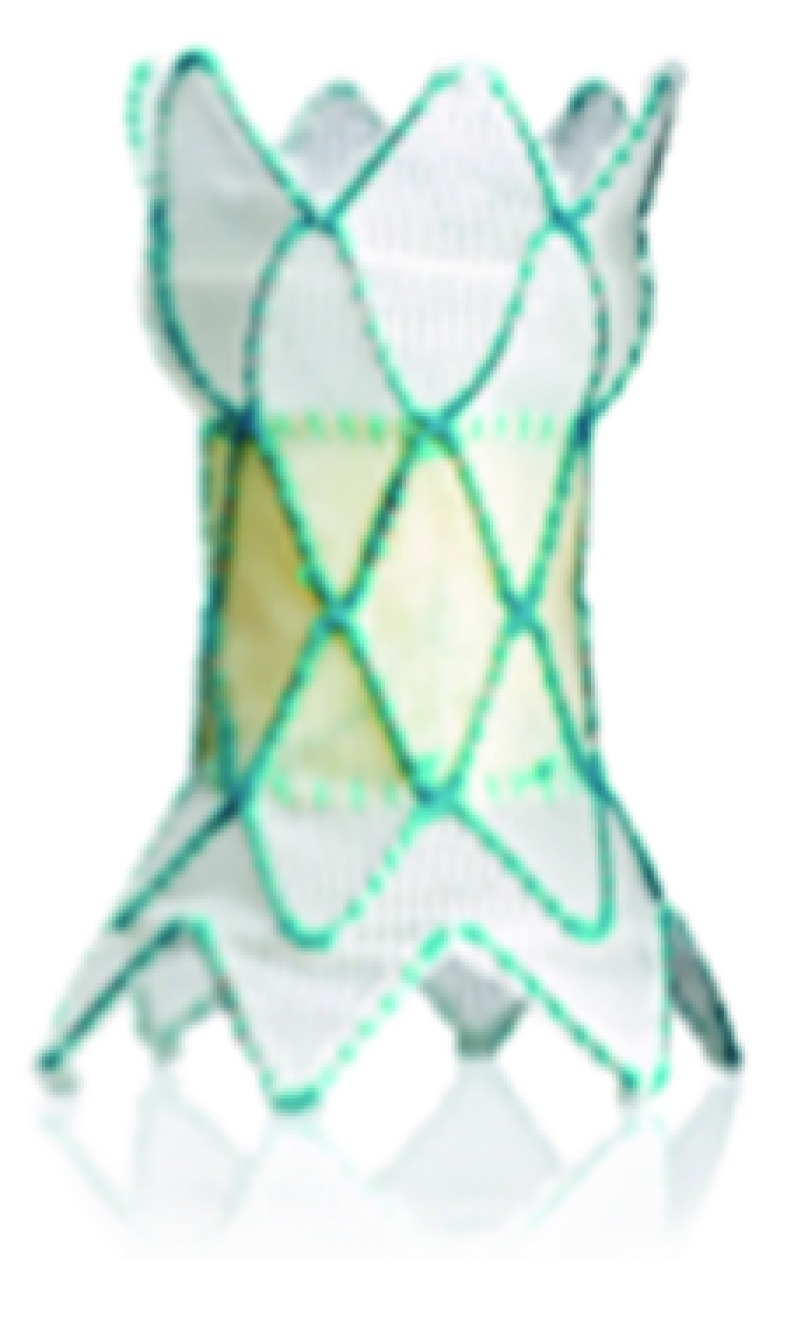
Harmony transcatheter pulmonary valve. The Medtronic Harmony transcatheter pulmonary valve (hTPV) system (Medtronic) has been specifically developed for implantation in non-uniform right ventricular outflow tracts after transannular patch repair of tetralogy of Fallot, building on Medtronic’s success with the Melody valve.

The Medtronic Harmony transcatheter pulmonary valve (hTPV) system (Medtronic) was developed after a fairly extensive experience with the Medtronic Melody valve system ( Figure 3). It was developed for patients with severe pulmonary regurgitation after surgical repair of congenital heart disease without right ventricle to pulmonary artery conduit placement. The hTPV is a self-expanding transcatheter valve, whereas the Melody valve is a balloon-expandable valve.

Animal studies using the hTPV in an ovine model of pulmonary regurgitation have demonstrated that implantation of the valve is both feasible and efficacious, positively impacting on pulmonary regurgitant fraction, right ventricular volumes, and biventricular function ^7^. An international, multi-centre, prospective human feasibility study with strict inclusion and exclusion criteria is ongoing with the intention to demonstrate the feasibility of implanting the hTPV in humans and to assess its safety and performance ^8^. Early publications and short-term outcomes in the Harmony Feasibility study have been promising with good procedural success rates and favourable valve function at early follow-up ^9^.

## Pulsta valve

**Figure 4.**
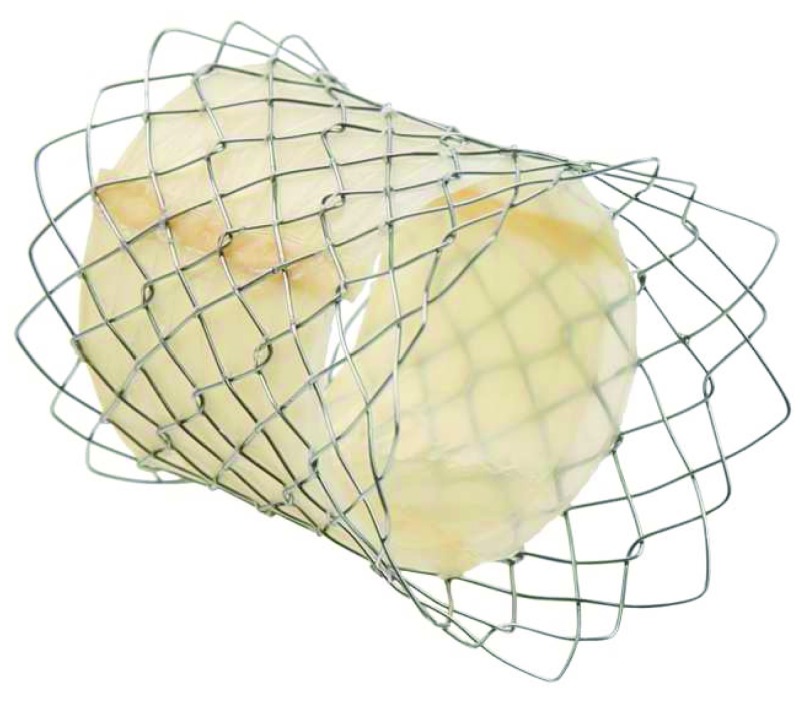
Pulsta valve. The Pulsta valve (TaeWoong Medical Co. Ltd) has been successfully implanted in a small number of human subjects.

The Pulsta valve (TaeWoong Medical Co., Ltd., Gimpo-si, Gyeonggi-do, South Korea) is a novel self-expanding transcatheter valve that was developed in South Korea ( Figure 4). It is composed of a nitinol stent covered in porcine pericardium. The valve is made of porcine pericardium and hand-sewn to the outer stent.

Preclinical animal studies have shown promising results ^10^, and the first published human case demonstrated that transcatheter implantation of this new self-expanding pulmonary valve in a patient with non-conduit pulmonary regurgitation was feasible with good immediate results ^11^.

## Hybrid surgical and transcatheter pulmonary valve implantation

Despite the advances in transcatheter therapies since the turn of the millennium, there remains a cohort of patients with pulmonary regurgitation, predominantly those who have undergone transannular patch repair of ToF, for whom a transcatheter approach to management is challenging for the available technology. The conventional approach to managing these patients is to undertake surgical valve implantation. Results of surgical pulmonary valve implantation to treat pulmonary regurgitation are generally excellent ^12^, and freedom from pulmonary valve reintervention at 5 years is around 95% ^13^.

However, these operations necessitate the use of cardiopulmonary bypass and cardioplegia and are associated with morbidity related to both this and the need for re-sternotomy. For patients at higher risk of complications, one potential treatment strategy might be a combined surgical and transcatheter approach to valve implantation ^14^. The No-React Injectable Bio Pulmonic (Bio Integral Surgical Inc., Toronto, ON, Canada) can be implanted in the RVOT after sternotomy and surgical plication of the outflow tract without the need for cardiopulmonary bypass ^15^. This reduces operation times and blood product use, but it remains to be seen whether this approach, avoiding cardiopulmonary bypass, results in clinically advantageous benefits when compared with conventional surgical pulmonary valve implantation.

## Conclusions

The management of pulmonary regurgitation after surgical repair of ToF has evolved over the last 20 years. Until recently, only about 25% of patients were suitable for transcatheter valves. With further developments in the technology, a majority of patients may be potentially treatable by transcatheter valve technology now and within the next decade. The number of patients in whom transcatheter valve therapy may be an option has expanded with the advent of novel valves designed specifically for use in the larger, non-conduit, outflow tracts (as opposed to fixed-dimension conduits). However, each of these valves is currently at varying stages of clinical trials, and it remains to be seen whether the promising early results translate into medium- and long-term outcomes comparable to those of the commercially available, smaller-diameter valves or surgical valve implantation or both. The hybrid surgical and transcatheter approach to pulmonary valve implantation is an interesting addition to the cardiologist’s and cardiac surgeon’s arsenal for these patients with complex anatomy. Ongoing studies of each of these approaches will hopefully provide support for their use and further benefit our patients.
